# Autologous Fat Grafting for the Treatment of a Painful Neuroma of the Hand: A Case Report and Review of Literature

**DOI:** 10.7759/cureus.10381

**Published:** 2020-09-11

**Authors:** Frank De Jongh, Sjaak Pouwels, Liang Tik Tan

**Affiliations:** 1 Plastic Surgery, Haaglanden Medisch Centrum, The Hague, NLD; 2 Intensive Care Medicine, Elisabeth-Tweesteden Hospital, Tilburg, NLD; 3 Plastic Surgery, Haaglanden Medical Center, The Hague, NLD

**Keywords:** autologous fat transfer, neuroma, neuro-in-continuity, plastic surgery, pain relief

## Abstract

Neuropathic pain caused by a neuroma can have a significant effect on daily life. Current surgical treatments include simple neuroma excision and proximal nerve stump relocation (into a muscle, vein, or bone). We describe a patient who presented with neuropathic pain, restricted to the dorsum of the right hand, and numbness of the dorsum of the radial half of the middle finger. The patient is a right-handed architect and due to the trauma could no longer shake hands for fear of pain. Her Tinel’s test was strongly positive. In 2015, she was diagnosed with a neuroma-in-continuity of the third digital nerve originating from the superficial branch of the radial nerve. At the time she was treated with an on-site Naropin injection and hand rehabilitation therapy, which ultimately alleviated the pain. Three years later she presented with pain progression whereupon we treated her exclusively with AFT. The patient was followed up for 12 weeks after the operation; the pain completely disappeared and the patient could shake hands again. After one year, she was still pain-free.

AFT is a new technique for the treatment of persistent neuropathic pain and numbness in the hand caused by blunt-trauma neuroma. Autologous fat grafting is a safe, effective, minimally invasive, and innovative therapeutic approach for the management of painful neuromas.

## Introduction

A neuroma is a benign tumour or growth of the nerve tissue which may occur after sharp, blunt, or traction trauma to the nerve [1, 2]. When damage to the nerve is greater, axon fascicles escape out of the damaged perineurium and form a painful swelling. When the nerve is only partially injured, a neuroma-in-continuity can form [1, 2]. A traumatic neuroma may be characterized by paraesthesia such as an electric pain, or a painful tingling sensation (hyperaesthesia), or a feeling of numbness [2]. Diverse options are available for the treatment of neuromas [3]. Its conservative treatment involves pharmacotherapy, which has to be used over the long term, has many side effects, and provides only short-term relief [4-6]. Injections of ethanol, lidocaine, or hormones can also be given as nerve blocks or trigger point injections [7, 8]. Surgical treatment of (painful) neuromas is controversial and has been highly debated, especially simple neuroma excision, which has a high recurrence of pain and provides unsatisfying results [9, 10]. Recently, autologous fat transfer (AFT) has been used for the treatment of neuropathic pain caused by neuromas, burns, scars, and post-mastectomy syndrome [11-18]. It has proven to be beneficial in all of these situations.

Here we present a patient with a compressed and painful neuroma in the back of the hand. We treated it with AFT therapy which decreased the neuropathic pain and alleviated sensory loss in the surrounding area of the skin innervated by the same nerve.

## Case presentation

**Figure 1:**
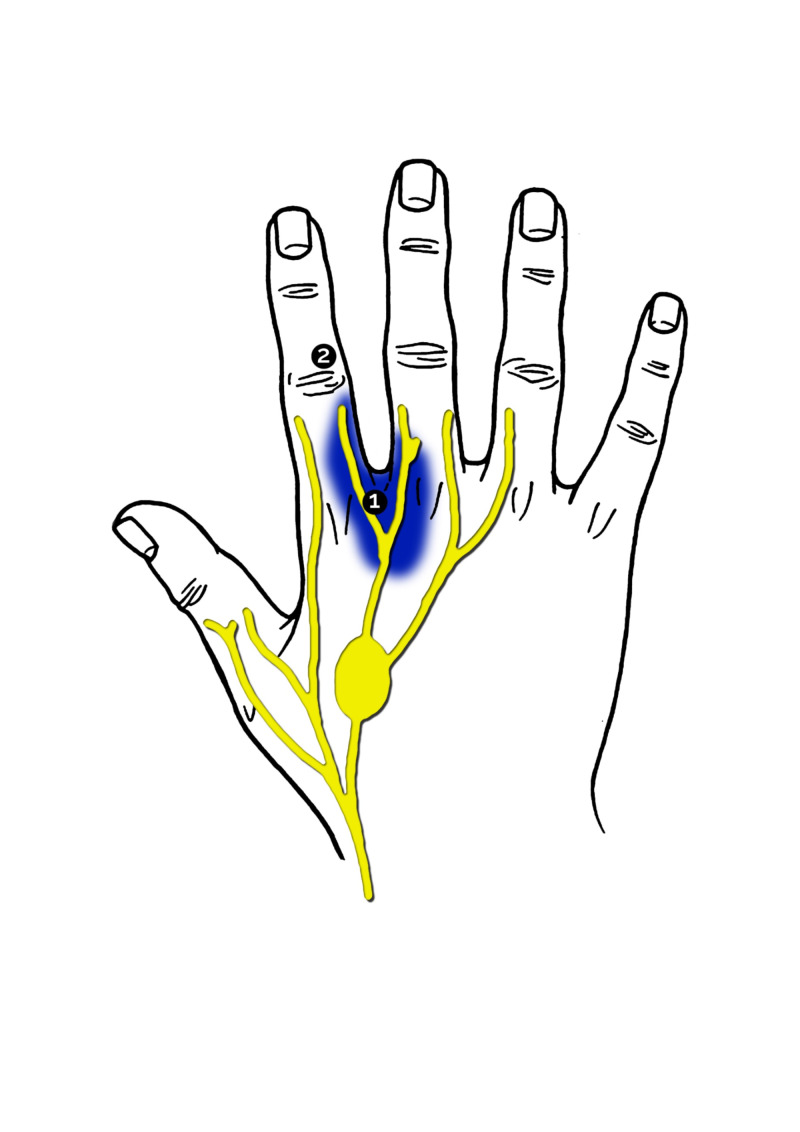
Area experiencing neuroma pain and numbness. The encircled numbers mark the areas that underwent sensorimotor rehabilitation. This figure was designed and developed by G.B. de Zeeuw

A 48-year-old female presented to us with neuropathic pain, restricted to the dorsum of the right hand, and numbness of the dorsum of the second web space and ulnar half of the index finger (Figure 1). The patient had experienced blunt trauma to this region of her hand in May 2015.

The patient complained of electric pain and painful tingling when the site on the back of the hand was touched. The patient is a right-handed architect and due to the trauma could no longer shake hands for fear of pain. She was diagnosed with neuroma-in-continuity of the third digital nerve originating from the superficial branch of the radial nerve. She was first treated with a trigger point injection of Naropin; this proved to be ineffective. She was then referred to a hand therapist to start sensorimotor rehabilitation, which alleviated the painful neuroma. Rehabilitation of the hypoaesthetic territory of the dorsum of the index finger was also performed and was ultimately successful. Aesthesiography results are shown in Figure 1 and Table 1.

**Table 1:** Aesthesiogram of the hypoaesthetic patient. The score for an area is equivalent to the amount of mass (in grams) which elicits a sensation when placed onto that area. In normal areas, sensation starts at 0.2 grams.

Date	Score (g)	
	area 1	area 2
25-06-2015	2.8	X
17-07-2015	1.8	X
24-09-2015	0.7	X
30-10-2015	0.55	0.8
04-12-2015	0.2	0.25

Three years later, she presented with worsening neuropathic pain and numbness in the previously treated areas of her hand. The pain was restricted to the dorsal surface of MCP2/3 (the second and third metacarpophalangeal joints). In contrast, the recurrent numbness was restricted to the dorso-ulnar surface of the proximal phalanx of the right hand. The pain was not related to posture, nor did it radiate to another site, and it only occurred in response to touch. Her Tinel’s test was strongly positive. There was no loss of motor function. She was scheduled for AFT and the surgery was performed under general anaesthesia. The donor site (abdomen) was infiltrated with 0.9% NaCl and 0.001 mg/ml adrenaline and the adipose tissue was harvested. Only 2 cm3 of the lipoaspirate was then injected subcutaneously into the pre-operatively marked area of the hand (Figure 1). After the operation, a pressure bandage was applied to her hand and the patient was advised to keep it held high. The patient also wore a corset around her abdomen to maintain pressure on the donor site for two weeks. The patient was monitored for 12 weeks after the operation. The neuroma pain completely disappeared and the patient could shake hands again. Also, the numbness on the dorso-ulnar surface of the proximal phalanx was cured. After one year she was still free of pain and numbness in her hand and therefore satisfied with the results.

## Discussion

We found that AFT proved effective in decreasing neuropathic pain and in alleviating hyposensitivity in the area around the neuroma.

A neuroma is either a benign tumour or growth of nerve tissue which may occur after any sharp, blunt, or traction trauma to the nerve [1, 2]. Two hypotheses are most widely accepted regarding the process of neuroma formation: 1) after greater nerve trauma, axon fascicles escape out of the damaged perineurium and form a painful swelling, while 2) partial nerve injury can lead to the formation of a neuroma in continuity [2]. Clinical sequelae can be characterised by paraesthesia and/or hyperesthesia and/or feeling of numbness [2].

As discussed earlier, either pharmacotherapy or surgical options can be considered for treating neuroma pain [3]. For pharmacotherapy, drugs like antidepressants, NSAIDs, opiates, α-receptor blockers, lidocaine, and antispasmodic drugs are prescribed. However, these drugs often need to be used over the long term, despite their side effects, since they only provide temporary pain relief [4-6]. Injections of ethanol, lidocaine, or hormones can also be given as nerve blocks or as trigger point injections [7, 8].

Surgical treatment of (painful) neuromas is highly debated, especially the simple neuroma excision procedure, which is associated with high recurrence of pain and unsatisfying results [9, 10]. In contrast, targeted nerve implantation (TNI) does have a role in the prevention of recurrent end-neuromas, which occur mostly in amputee patients [19]. The relocation of the nerve end into a muscle, vein, or even a bone has been described previously [11-20].

For sensorimotor rehabilitation, aesthesiography or allodynography are performed to quantify neuropathic pain as described by C. J. Spicher [1, 2, 11-20]. Rehabilitation of hypoaesthesia or allodynia is possible because of the neuroplasticity of the somatosensory system. It is believed that mechanical allodynia masks hypoaesthesia. Thus when the zone of allodynia is treated successfully, the underlying zone hypoaesthesia also gets remedied. The increasing sensation will subsequently decrease neuropathic pain [1, 2, 11-20].

A potential bias of our study is that the patient did not undergo aesthesiography when she presented to us in 2018, therefore her symptoms and the effect of AFT could not be quantified.

Recently, autologous fat transfer (AFT) has been used for the treatment of neuropathic pain caused by neuromas, burns, scars, and post-mastectomy syndrome [11-18]. Its benefits are both mechanical and biological. The adipose tissue creates a gliding layer which functions as a protective wrap and allows free excursion of the nerves [19, 20]. The biological benefits include neoangiogenesis [11-18], inflammatory response modulation, and prevention of scar adhesions [11-20].

## Conclusions

Neuroma pain in the right hand was successfully treated with autologous fat transfer. Autologous fat transfer has been used in a variety of other clinical situations like burns, scars, and post-mastectomy syndrome. Although it seems to have beneficial results, further research is needed to substantiate these findings.
